# Rangoon Lunatic Asylum

**Published:** 1901-10-19

**Authors:** 


### RANGOON LUNATIC ASYLUM.

During the year 1899 an attempt had been made to reorganise the Rangoon Lunatic Asylum. The deputy-superintendent, Mr. Donaldson, was replaced by a medical man, which, it was hoped, might prove to be a move in the right direction. Unfortunately, the change has not worked satisfactorily, and fresh arrangements have to be made, which is the more a matter of regret because the sub-overseer has been reported unfit for his duties, and a new man will be necessary. Happily, some of the alterations also may be termed improvements viz., the appointment of a matron to take charge of the female enclosure, and an increase in the number of warders, five male and three female attendants having been added to the staff. There was a rise in the number of inmates received during the twelve months ended December, 1899, the total being 462 as against 345 in the previous year. The result of this increase has occasioned overcrowding on the female side of the asylum. The accommodation is for 30 patients only. At one time there were 52 women in the asylum, and owing to the fact that a new cottage destined to hold twelve more lunatics, where building operations were commenced some time back, is not yet ready for use, the objectionable practice of confining some of the older females in the male cells at night has to be continued. There were 117 admissions during the year 1899, as compared with 88 in the previous twelve months. Of these only 26 were criminal lunatics, six less than in 1898, so that the large increase was on the civil side These figures appear to point to a corresponding increase in insanity in the province, but this is not thought to be the case. There are two causes which have tended to raise the number of admissions, firstly, that as years go on and peace and order prevail, lunatics, who of old would have been neglected and left to themselves, are now forwarded to the asylum for treatment. Also large numbers of foreigners are constantly arriving at the large ports for work in the rice trade. Many of these from sickness (principally syphilis), drink, and owing to the use of drugs become unfit for work, find themselves stranded in Burma far from home and friends, and so lose the few wits they may have originally possessed. They are then brought to the asylum as "wandering lunatics," generally in a half-starved, deplorable condition. It is this class of lunatics who cause the heavy death rate noticeable in the statistics. Thirty of the 462 patients died during the year, the health of 21 of these having been classified upon admission as "bad," "poor," or "indifferent." It is satisfactory to note that, notwithstanding the high ratio of mortality, there is a decrease from the figures of the preceding year, which in their turn were lower than in 1897. There was no death from smallpox, cholera, or chicken-pox, the asylum having enjoyed complete immunity from epidemics, with the exception of influenza. There were six cases of pneumonia, which have not arisen from the want of warm clothing, as it was given out to the patients at once when the cold season commenced. As the asylum is not yet able to be conducted on the most modern European methods with night attendants constantly in the wards where the lunatics sleep, it is impossible to prevent clothing being discarded by the most pronounced cases of lunacy, and this inevitably leads to chills. With regard to the number of discharges, out of the 67 recorded eight were civil lunatics transferred to the Madras Asylum. As to patients restored to health, the percentage is very low, 7 per cent. for mania and 3 per cent. for melancholia; but it must be taken into consideration that in Burma the police do not interfere till a patient's derangement is so advanced that he becomes unmanageable. Then the case is taken to the asylum. Four lunatics succeeded in escaping during 1899, two of whom are still at liberty. Strangely enough, one of these had been an inmate of the asylum for eighteen years, and had always been quiet and harmless, though of poor physique. So cleverly did he make his escape that his method of doing so is still unknown. There is a decrease in the total expenditure in 1899 of R.7,084, which is chiefly because the warders' suits have during the year been manufactured and made in the asylum instead of being bought as before from the Insein Jail, because the receipts on sales of flowers and vegetables from the gardens is nearly double what it was, and because the lunatics have been largely employed in the manual labour of the institution. Fourteen theatrical performances have been held for the amusement of the patients during the year. Burmese football has been played, and cards, Burmese dominoes and Burmese draughts, as well as a small library with two daily papers—one in English, one in Burmese—have been supplied for the use of the inmates and seem to be much appreciated.

